# Effect of dapagliflozin on kidney and cardiovascular outcomes by baseline KDIGO risk categories: a post hoc analysis of the DAPA-CKD trial

**DOI:** 10.1007/s00125-022-05694-6

**Published:** 2022-04-21

**Authors:** Simke W. Waijer, Priya Vart, David Z. I. Cherney, Glenn M. Chertow, Niels Jongs, Anna Maria Langkilde, Johannes F. E. Mann, Ofri Mosenzon, John J. V. McMurray, Peter Rossing, Ricardo Correa-Rotter, Bergur V. Stefansson, Robert D. Toto, David C. Wheeler, Hiddo J. L. Heerspink

**Affiliations:** 1 Department of Clinical Pharmacy and Pharmacology, University of Groningen, University Medical Center Groningen, Groningen, the Netherlands; 2 Department of Medicine, Division of Nephrology, University Health Network and University of Toronto, Toronto, Canada; 3 Departments of Medicine and Epidemiology and Population Health, Stanford University School of Medicine, Stanford, CA USA; 4 Late-Stage Development, Cardiovascular, Renal and Metabolism, BioPharmaceuticals R&D, AstraZeneca, Gothenburg, Sweden; 5 KfH Kidney Center Munich, Germany, and Department of Medicine 4, University of Erlangen-Nürnberg, Erlangen, Germany; 6 Diabetes Unit, Department of Internal Medicine, Hadassah Hebrew University Hospital, Jerusalem, Israel; 7 Institute of Cardiovascular and Medical Sciences, University of Glasgow, Glasgow, UK; 8 Steno Diabetes Center Copenhagen, Gentofte, Denmark; 9 Department of Clinical Medicine, University of Copenhagen, Copenhagen, Denmark; 10 The National Medical Science and Nutrition Institute Salvador Zubiran, Mexico City, Mexico; 11 Department of Internal Medicine, UT Southwestern Medical Center, Dallas, TX USA; 12 Department of Renal Medicine, University College London, London, UK; 13 The George Institute for Global Health, Sydney, NSW Australia

**Keywords:** Albuminuria, Dapagliflozin, eGFR, KDIGO risk categories, Kidney outcome, SGLT2 inhibitor

## Abstract

**Aims/hypothesis:**

In the Dapagliflozin and Prevention of Adverse Outcomes in Chronic Kidney Disease (DAPA-CKD) trial, dapagliflozin reduced the risks of progressive kidney disease, hospitalised heart failure or cardiovascular death, and death from all causes in patients with chronic kidney disease (CKD) with or without type 2 diabetes. Patients with more severe CKD are at higher risk of kidney failure, cardiovascular events and all-cause mortality. In this post hoc analysis, we assessed the efficacy and safety of dapagliflozin according to baseline Kidney Disease Improving Global Outcomes (KDIGO) risk categories.

**Methods:**

DAPA-CKD was a double-blind, placebo-controlled trial that randomised patients with an eGFR of 25–75 ml min^−1^ [1.73 m]^−2^ and urinary albumin/creatinine ratio (UACR) of ≥22.6 and <565.0 mg/mmol (200–5000 mg/g) to dapagliflozin 10 mg/day or placebo. The primary endpoint was a composite of ≥50% reduction in eGFR, end-stage kidney disease (ESKD), and death from a kidney or cardiovascular cause. Secondary endpoints included a kidney composite (≥50% reduction in eGFR, ESKD and death from a kidney cause), a cardiovascular composite (heart failure hospitalisation or cardiovascular death), and death from all causes. We used Cox proportional hazards regression analyses to assess relative and absolute effects of dapagliflozin across KDIGO risk categories.

**Results:**

Of the 4304 participants in the DAPA-CKD study, 619 (14.4%) were moderately high risk, 1349 (31.3%) were high risk and 2336 (54.3%) were very high risk when categorised by KDIGO risk categories at baseline. Dapagliflozin reduced the hazard of the primary composite (HR 0.61; 95% CI 0.51, 0.72) and secondary endpoints consistently across KDIGO risk categories (all *p* for interaction >0.09). Absolute risk reductions for the primary outcome were also consistent irrespective of KDIGO risk category (*p* for interaction 0.26). Analysing patients with and without type 2 diabetes separately, the relative risk reduction with dapagliflozin in terms of the primary outcome was consistent across subgroups of KDIGO risk categories. The relative frequencies of adverse events and serious adverse events were also similar across KDIGO risk categories.

**Conclusion/interpretations:**

The consistent benefits of dapagliflozin on kidney and cardiovascular outcomes across KDIGO risk categories indicate that dapagliflozin is efficacious and safe across a wide spectrum of kidney disease severity.

**Trial registration:**

ClinicalTrials.gov NCT03036150.

**Funding:**

The study was funded by AstraZeneca.

**Graphical abstract:**

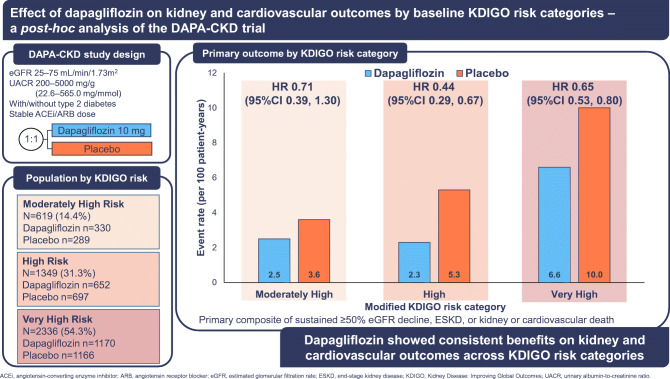

**Supplementary Information:**

The online version contains peer-reviewed but unedited supplementary material available at 10.1007/s00125-022-05694-6.

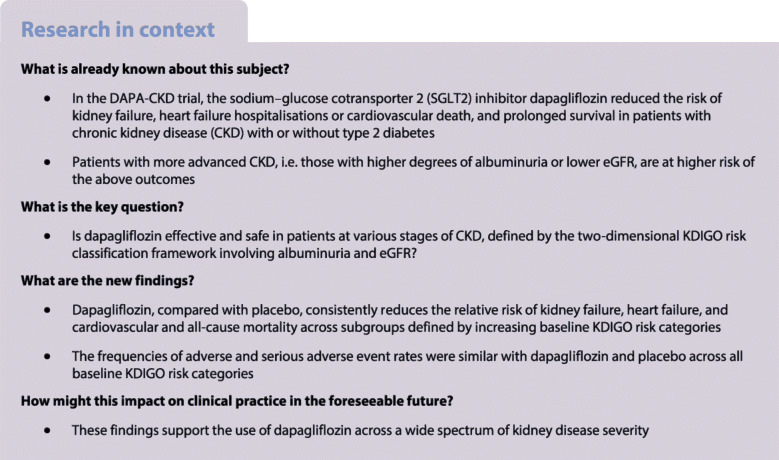



## Introduction

Sodium–glucose cotransporter 2 (SGLT2) inhibitors were originally developed as oral glucose-lowering drugs. Previous studies showed that the HbA_1c_-lowering efficacy of SGLT2 inhibitors is attenuated or absent in patients with reduced eGFR [1, 2]. Due to the lack of glycaemic efficacy, it was assumed that SGLT2 inhibitors would not prevent micro- and macrovascular complications in patients with diabetes and reduced eGFR [2]. However, several clinical trials demonstrated that SGLT2 inhibitors prevent progression of chronic kidney disease (CKD), kidney failure, and cardiovascular events in patients with CKD [3–5]. The Canagliflozin and Renal Events in Diabetes With Established Nephropathy Clinical Evaluation (CREDENCE) trial demonstrated these clinical benefits in patients with type 2 diabetes and CKD [3]. The Dapagliflozin and Prevention of Adverse Outcomes in Chronic Kidney Disease (DAPA-CKD) trial extended these findings to a broader population of patients with CKD, with or without type 2 diabetes, and the results were independent of the degree of glycaemic control [4, 6, 7].

Higher albuminuria and lower eGFR are well-established predictors of kidney failure and cardiovascular events, and form the foundation of the Kidney Disease Improving Global Outcomes (KDIGO) CKD disease classification system [8]. The inclusion of both albuminuria and eGFR in the KDIGO CKD classification system allows cardiovascular and kidney risk categorisation based on combined albuminuria and eGFR assessment. Whether the clinical benefits of the SGLT2 inhibitor dapagliflozin in patients with CKD are generalisable to patients at various stages of CKD as defined by baseline KDIGO classification is unknown. In addition, whether the KDIGO CKD classification can be used to identify patients who may derive greater absolute benefit from dapagliflozin, or whether there are subpopulations without such benefits, has not been determined, but may guide treatment decisions in clinical practice.

In this post hoc analysis of the DAPA-CKD trial, we examined the efficacy and safety of dapagliflozin according to baseline KDIGO risk categories among patients with and without type 2 diabetes.

## Methods

### Study design and participants

DAPA-CKD was a randomised, double-blind, placebo-controlled multicentre, international trial conducted in 21 countries at 386 study sites. The study design and the primary results have been published previously [4, 9]. Briefly, we enrolled adult patients with CKD with and without type 2 diabetes who were ≥18 years of age with eGFR ≥25 and <75 ml min^−1^ [1.73 m]^−2^ and urinary albumin/creatinine ratio (UACR) ≥22.6 and <565.0 mg/mmol (≥200 and <5000 mg/g). Patients with type 1 diabetes, polycystic kidney disease, lupus nephritis or anti-neutrophil cytoplasm antibodies (ANCA)-associated vasculitis, as well as those receiving immunotherapy for primary or secondary kidney disease within 6 months prior to enrolment, were excluded. All eligible patients were receiving treatment with a stable dose of an ACE inhibitor or angiotensin receptor blocker for ≥4 weeks prior to randomisation unless there was a documented intolerance to these drugs. The trial protocol was approved by a central or local ethics committee at each trial site, and all participants provided written informed consent. This study was registered on ClinicalTrials.gov (NCT03036150) and posted online on 30 January 2017, prior to enrolment of the first participant.

### Randomisation and follow-up

We randomly assigned eligible participants to receive dapagliflozin 10 mg once daily or matching placebo. The study drug was to be continued until the occurrence of diabetic ketoacidosis, pregnancy, receipt of disallowed therapy, or study completion. Following randomisation, in-person study visits were performed after 2 weeks, at 2, 4 and 8 months, and at 4-month intervals thereafter. At each follow-up visit, we recorded vital signs, sent blood and urine samples for laboratory assessment, and collected information on potential study endpoints, adverse events, concomitant therapies and study drug adherence.

### Classification of KDIGO risk categories

**Fig. 1:**
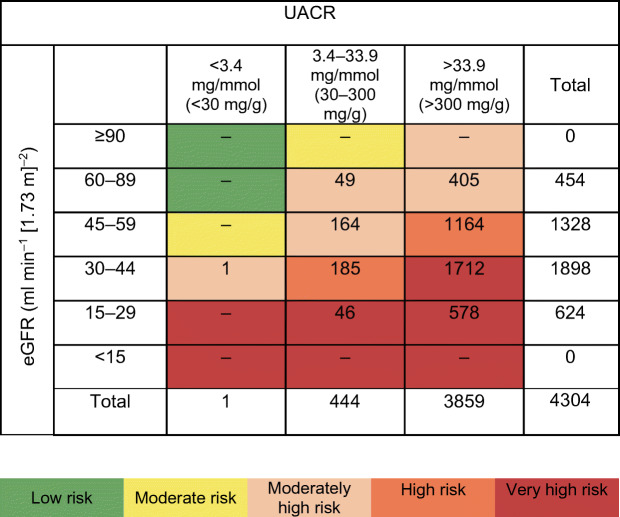
Number of patients by modified KDIGO risk categories. The KDIGO risk categories refer to a modified categorisation that includes an additional category of moderately high risk

We categorised patients according KDIGO risk categories based on their eGFR and albuminuria level. As many DAPA-CKD participants were categorised in the original very high KDIGO risk category, we created an additional category in order to stratify the cohort across three subgroups with approximately equal sample sizes. We therefore defined the following categories in this study (Fig. 1): moderately high risk (baseline eGFR 30–44 ml min^−1^ [1.73 m]^−2^ and UACR <3.4 mg/mmol [<30 mg/g]; or baseline eGFR 45–89 ml min^−1^ [1.73 m]^−2^ and UACR 3.4–33.9 mg/mmol [30–300 mg/g]; or baseline eGFR >60 ml min^−1^ [1.73 m]^−2^ and UACR >33.9 mg/mmol [>300 mg/g]); high risk (baseline eGFR 30–44 ml min^−1^ [1.73 m]^−2^ and UACR 3.4–33.9 mg/mmol [30–300 mg/g]; or baseline eGFR 45–59 ml min^−1^ [1.73 m]^−2^ and UACR >33.9 mg/mmol [>300 mg/g]); and very high risk (baseline eGFR <30 ml min^−1^ [1.73 m]^−2^ and UACR 3.4–33.9 mg/mmol [30–300 mg/g]; or baseline eGFR <45 ml min^−1^ [1.73 m]^−2^ and UACR >33.9 mg/mmol [>300 mg/g]). The term ‘KDIGO risk categories’ used in this manuscript refers to this modified categorisation.

### Outcomes

The primary endpoint was a composite of a sustained ≥50% decline in eGFR (confirmed by a second serum creatinine measurement after at least 28 days), onset of end-stage kidney disease (ESKD, defined as maintenance dialysis for more than 28 days, kidney transplantation, or eGFR <15 ml min^−1^ [1.73 m]^−2^ confirmed by a second measurement after at least 28 days), or death from a kidney or cardiovascular cause. Secondary outcomes were, in hierarchical order: (1) a kidney-specific endpoint defined in the same way as the primary outcome but excluding death from a cardiovascular cause; (2) a cardiovascular composite endpoint of cardiovascular death or hospitalisation for heart failure; and (3) all-cause mortality. An independent blinded event adjudication committee assessed all clinical endpoints using rigorous pre-specified endpoint definitions.

### Statistical analysis

The overall analytic approach, power calculation, and pre-specified statistical analysis plan have been published previously [9]. Briefly, we performed time-to-event analyses using a Cox proportional hazards regression stratified for randomisation factors (diabetes status and UACR) and adjusted for baseline eGFR. We calculated the HRs and 95% CI from the model parameter coefficients and standard errors, respectively. All analyses presented here followed the intention-to-treat principle. For the purpose of the current analysis, we evaluated the primary and secondary efficacy endpoints in patients stratified by the modified KDIGO risk categories. The effects of dapagliflozin by KDIGO risk categories were separately assessed in patients with and without type 2 diabetes. We avoided including redundant terms for the randomisation strata and subgroup factors in the statistical models when the two coincided. We compared results across KDIGO risk groups by including a multiplicative interaction term between randomised treatment group and KDIGO risk categories. We also performed exploratory analyses stratified by UACR and eGFR separately. In these analyses, we categorised patients based on baseline UACR into three categories (≤113.0 mg/mmol [≤1000 mg/g], >113.0 to ≤395.5 mg/mmol [>1000 to ≤3500 mg/g] and >395.5 mg/mmol [>3500 mg/g]) and categorised patients based on eGFR into subgroups (<30 ml min^−1^ [1.73 m]^−2^; ≥30 to <45 ml min^−1^ [1.73 m]^−2^; ≥45 ml min^−1^ [1.73 m]^−2^). In additional analyses, to investigate whether the effect of treatment varies by levels of baseline UACR, interaction was tested between treatment and UACR (continuous) separately in patients with and without type 2 diabetes. To allow for non-linearity of the effect of treatment across levels of baseline UACR, UACR was transformed by fractional polynomials. For the time-to-event analyses, we assessed the proportional hazards assumption using the Akaike’s information criterion and Schoenfeld residuals test.

We analysed the effects of dapagliflozin on the mean on-treatment eGFR slope by fitting a two-slope mixed effects linear spline model (with a knot at week 2) with a random intercept and random slopes for treatment. The model included fixed effects for treatment, baseline KDIGO category, stratification factors (diabetes status) and a continuous, fixed covariate for time-to-visit. To determine eGFR slopes for the KDIGO risk subgroup, we added to the model all possible interaction terms for treatment effect, KDIGO risk subgroup and time-to-visit, assuming an unstructured variance/covariance matrix. The mean total slope was computed as a weighted combination of the acute and chronic slopes, to reflect the mean rate of eGFR change until the last on-treatment visit. We also presented the pattern of change in mean eGFR using a restricted maximum-likelihood repeated-measures approach. This latter analysis included fixed effects of treatment, visit, treatment-by-visit interaction and treatment-by-KDIGO risk subgroup interaction. We added interaction terms among KDIGO risk subgroup, visit and treatment assignment to assess the change in eGFR within each subgroup. We considered *p* values <0.05 to be statistically significant, and all analyses were performed using Stata version 14.2 (StataCorp, USA) or R version 4.0.2 (R Foundation).

## Results

The DAPA-CKD trial included 4304 patients, who were randomly assigned to dapagliflozin (*n* = 2152) or placebo (*n* = 2152). The mean age at baseline was 61.8 years (SD 12.1), 1425 patients were female (33.1%), the mean eGFR was 43.1 ml min^−1^ [1.73 m]^−2^ (SD 12.4), and the median UACR was 107.2 mg/mmol (IQR 53.9–213.0) (949 mg/g [IQR 477–1885]); 2906 patients had type 2 diabetes (67.5%) and 1398 patients did not have diabetes (32.5%).

When categorised by baseline KDIGO risk categories, 619 patients (14.4%) were moderately high risk, 1349 (31.3%) were at high risk, and 2336 (54.3%) were very high risk (Fig. 1). Patients in the highest KDIGO risk category were more likely to have higher systolic blood pressure and lower HbA_1c_ compared with those with lower KDIGO risk (Table 1).

**Table 1:** Baseline characteristics by baseline KDIGO risk category

	KDIGO risk categories			*p* value^a^
	Moderately high risk	High risk	Very high risk	
Number of participants, *n* (%)	619 (14.4)	1349 (31.3)	2336 (54.3)	
Mean age, years (SD)	61.4 (11.6)	62.0 (12.0)	61.9 (12.3)	0.54
Female sex, *n* (%)	203 (32.8)	417 (30.9)	805 (34.5)	0.09
Race, *n* (%)				0.001
White	355 (57.3)	698 (51.7)	1237 (52.9)	
Black	32 (5.2)	55 (4.1)	104 (4.4)	
Asian	164 (26.5)	490 (36.3)	813 (34.8)	
Other	68 (11.0)	106 (7.9)	182 (7.8)	
Mean BMI, kg/m^2^ (SD)	29.9 (6.1)	29.4 (6.0)	29.5 (6.2)	0.17
Current smoker, *n* (%)	86 (13.9)	192 (14.2)	306 (13.1)	0.86
BP, mmHg				
Systolic	136.4 (16.2)	136.3 (16.9)	137.7 (17.9)	0.047
Diastolic	77.6 (9.7)	77.8 (10.4)	77.3 (10.7)	0.47
eGFR, ml min^−1^ [1.73 m]^−2^	62.7 (8.6)	49.5 (6.3)	34.2 (5.9)	<0.001
HbA_1c_, mmol/mol	57 (20.8)	54 (19.7)	52 (17.5)	<0.001
HbA_1c_, %	7.4 (1.9)	7.1 (1.8)	6.9 (1.6)	<0.001
Haemoglobin, g/l (SD)	134 (18)	132 (18)	125 (18)	<0.001
Median UACR, mg/mmol (IQR)	58.4 (30.0–147.4)	93.1 (47.0–193.1)	126.9 (68.6–242.8)	<0.001
Median UACR, mg/g (IQR)	517 (265–1304)	824 (416–1709)	1123 (607–2149)	<0.001
Type 2 diabetes, *n* (%)	470 (75.9)	915 (67.8)	1521 (65.1)	<0.001
Cardiovascular disease, *n* (%)	248 (40.1)	492 (36.5)	870 (37.2)	0.30
Medications				
ACE inhibitor/ARB, *n* (%)	613 (99.0)	1314 (97.4)	2247 (96.2)	0.001
Diuretics, *n* (%)	234 (37.8)	538 (39.9)	1110 (47.5)	<0.001
GLP-1 receptor agonists^b^, *n* (%)	20 (4.3)	31 (3.4)	71 (4.7)	0.31
Mineralocorticoid receptor antagonists, *n* (%)	24 (3.9)	78 (5.8)	127 (5.4)	0.20
Statin, *n* (%)	245 (39.6)	467 (34.6)	886 (37.9)	0.05

^a^Statistically significant differences across the three baseline KDIGO risk categories

^b^Only in patients with diabetes (*n* = 2906)

ARB, angiotensin receptor blocker; GLP-1, glucagon-like peptide 1

### Rates of the primary and secondary endpoints by baseline KDIGO risk

**Fig. 2:**
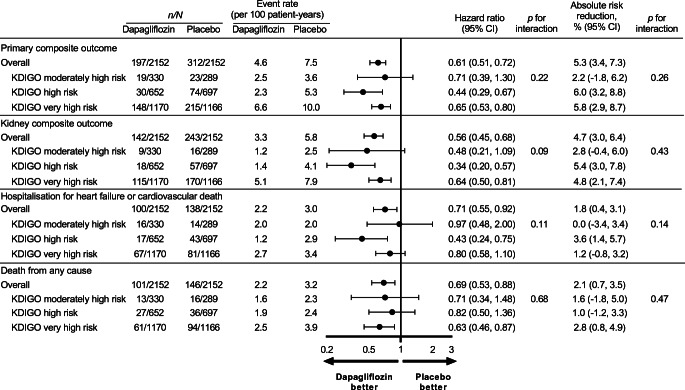
Relative effect and absolute risk reduction for dapagliflozin on the primary and secondary outcomes across different subgroups of KDIGO risk categories. The primary composite outcome is a composite of an eGFR decline of ≥50%, ESKD or death from kidney or cardiovascular causes. The kidney composite outcome is a composite of an eGFR decline of ≥50%, ESKD or death from kidney causes

Patients with a higher KDIGO risk category experienced a higher event rate for kidney and cardiovascular events, in both the placebo and dapagliflozin treatment groups. For example, patients randomised to placebo with a very high KDIGO risk experienced an event rate of 10.0 events (95% CI 8.7, 11.4) per 100 patient-years for the primary composite endpoint, while the event rate among patients with moderately high risk was 3.6 (95% CI 2.4, 5.4) (Fig. 2). The rate of heart failure hospitalisation or cardiovascular death among patients randomised to placebo with very high KDIGO risk was 3.4 events (95% CI 2.8, 4.3) per 100 patient-years compared with 2.0 (95% CI 1.2, 3.5) in patients with a moderately high KDIGO risk (Fig. 2).

### Relative risk reduction by KDIGO risk categories

Compared with placebo, there was no evidence of heterogeneity by KDIGO risk categories when considering relative effects of dapagliflozin on the primary composite endpoint (HR 0.61; 95% CI 0.51, 0.72). The HR with dapagliflozin compared with placebo was 0.65 (95% CI 0.53, 0.80) in patients in the very high KDIGO risk category, 0.44 (95% CI 0.29, 0.67) among patients in the high KDIGO risk category, and 0.71 (95% CI 0.39, 1.30) among patients in the moderately high KDIGO risk category (Fig. 2, *p* for interaction 0.22). Similarly, there was no evidence of heterogeneity by KDIGO risk categories when considering relative effects of dapagliflozin on secondary endpoints by KDIGO risk categories (Fig. 2). There was no evidence against the proportional hazards assumption.

### Absolute risk reduction by KDIGO risk categories

Although there was no evidence of heterogeneity by KDIGO risk categories when considering the absolute effects of dapagliflozin (*p* for interaction 0.26), the absolute benefits for the primary endpoint were numerically higher in patients with very high KDIGO risk (5.8%; 95% CI 2.9, 8.7) compared with patients with moderately high KDIGO risk (2.2%; 95% CI −1.8, 6.2). Absolute benefits of dapagliflozin on the secondary kidney endpoint, hospitalisation for heart failure or cardiovascular death endpoint and all-cause death were consistent across KDIGO risk categories (all *p* for interaction >0.14; Fig. 2).

### Efficacy in patients with and without type 2 diabetes in various KDIGO risk categories

**Fig. 3:**
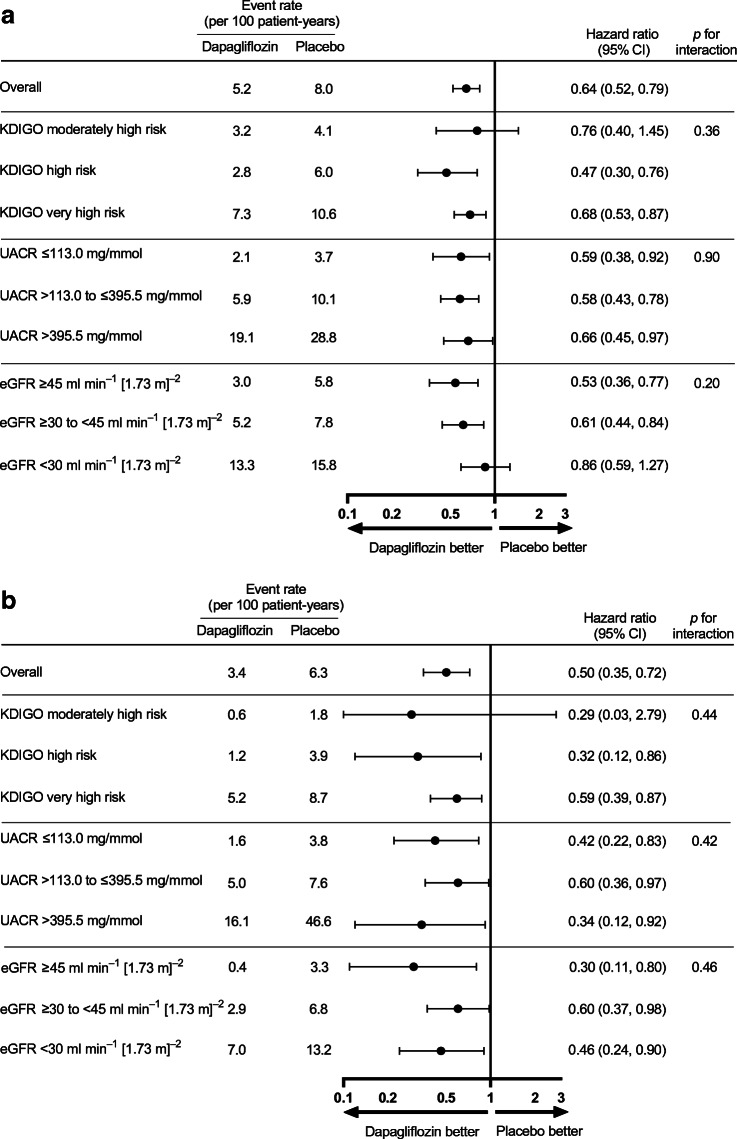
Effects of dapagliflozin on the primary composite outcomes across KDIGO risk categories and subgroups of UACR and eGFR in patients with diabetes (**a**) and without diabetes (**b**). The primary composite outcome is a composite of an eGFR decline of ≥50%, ESKD or death from kidney or cardiovascular causes

Analysing patients with and without type 2 diabetes separately, the effect of dapagliflozin compared with placebo on the primary composite endpoint was consistent across subgroups of baseline KDIGO risk categories in patients with and without type 2 diabetes (all *p* for interaction >0.36; Fig. 3). The effects of dapagliflozin on the three secondary endpoints were also consistent across KDIGO risk categories in patients with and without type 2 diabetes (electronic supplementary material [ESM] Fig. 1).

### Effects on eGFR slope by KDIGO risk category

**Fig. 4:**
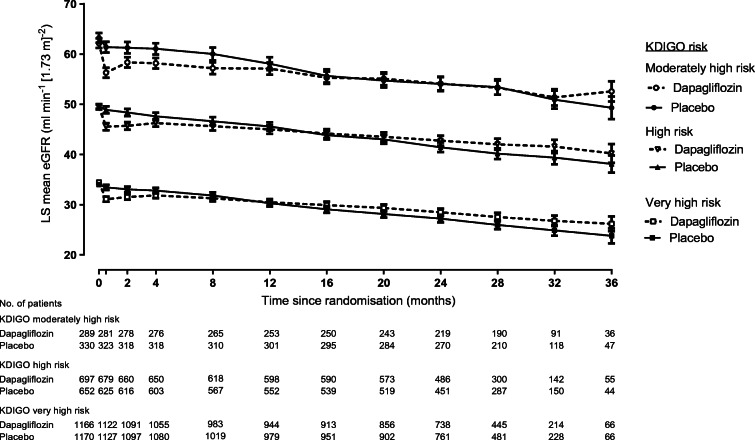
Effect of dapagliflozin on eGFR slope across KDIGO risk categories. LS, least squares. Data reported with 95% CI

The relative effect of dapagliflozin on eGFR slope was consistent across all subgroups of KDIGO risk categories (Fig. 4; ESM Fig. 2). For example, across KDIGO risk categories, initiation of dapagliflozin led to an acute drop in eGFR at week 2 that was consistent in moderately high, high and very high KDIGO risk categories. Placebo-subtracted differences in these groups were −2.7, −2.5 and −2.2 ml min^−1^ [1.73 m]^−2^, respectively (*p* for interaction 0.49). Thereafter, treatment with dapagliflozin compared with placebo led to a reduction in eGFR decline in all KDIGO risk categories with corresponding placebo-subtracted differences in chronic slope of 1.8 ml min^−1^ [1.73 m]^−2^ per year (95% CI 1.3, 2.2) in the very high risk category, 2.1 (95% CI 1.6, 2.7) in the high risk category, and 2.1 (95% CI 1.3, 2.9) in the moderately high risk category (*p* for interaction 0.34; Fig. 4; ESM Fig. 2). The effect of dapagliflozin on total slope was also consistent across KDIGO risk categories (ESM Fig. 2).

### Primary endpoint and secondary endpoints by baseline UACR and eGFR

Baseline characteristics for each baseline subgroup of UACR and eGFR are presented in ESM Table 1. The event rate for the primary endpoint was higher in patients with a lower baseline eGFR or higher UACR. For example, in patients randomised to placebo with an eGFR <30 ml min^−1^ [1.73 m]^−2^, the event rate per 100 patient-years for the primary endpoint was 14.9 (95% CI 12.1, 18.4) compared with 5.1 (95% CI 4.2, 6.2) in patients with an eGFR ≥45 ml min^−1^ [1.73 m]^−2^ (ESM Fig. 3).

Dapagliflozin consistently reduced the relative risks for the primary and all secondary endpoints across subgroups of UACR and eGFR (all *p* for interaction >0.10). Although the relative effects on the primary and kidney-specific secondary endpoint were consistent across baseline UACR subgroups, the absolute benefit on the primary and kidney-specific secondary endpoints was greater in subgroups with higher levels of albuminuria. The absolute effects of dapagliflozin on the primary composite and secondary endpoints were consistent across subgroups of eGFR (all *p* for interaction >0.36, ESM Fig. 3).

**Fig. 5:**
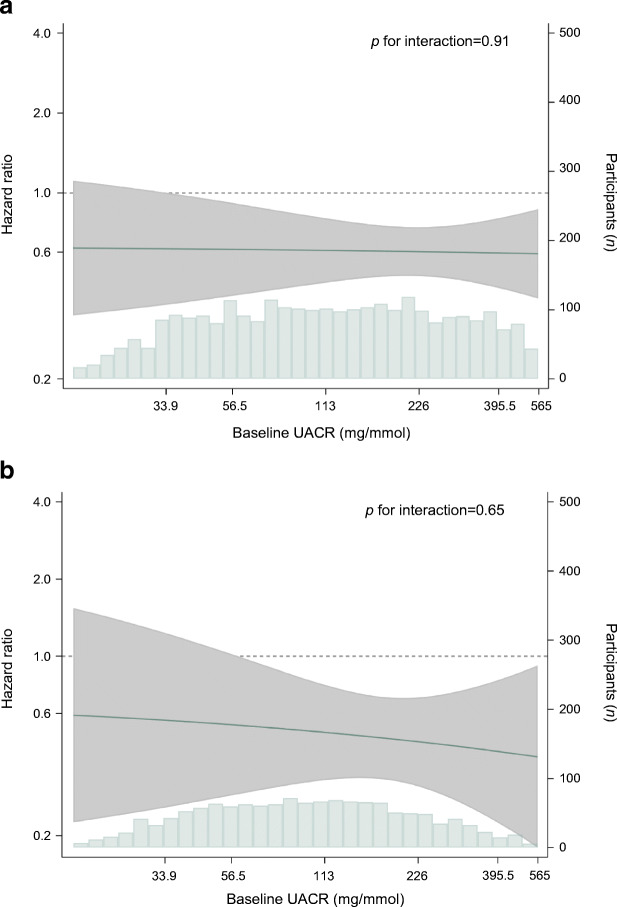
Effect of dapagliflozin on the primary composite outcome across baseline UACR levels in patients with diabetes (**a**) and without diabetes (**b**). The solid line indicates the HR for the primary outcome across baseline UACR, on the left *y*-axis plotted on a natural log scale; the shaded area indicates the 95% CI. The histogram shows the distribution of UACR at baseline, by number of participants on the right *y*-axis

When we analysed UACR as a continuous measure in patients with and without type 2 diabetes separately, we observed the benefit of dapagliflozin on the primary composite endpoint across a range of UACR levels (Fig. 5; *p* value heterogeneity 0.91 and 0.65, respectively).

There were 454 patients with an eGFR ≥60 ml min^−1^ [1.73 m]^−2^, of whom 33 experienced a primary composite endpoint. A total of 445 patients had UACR ≤33.9 mg/mmol (≤300 mg/g), of whom 18 experienced a primary composite endpoint. Although the number of patients and events in the groups with eGFR ≥60 ml min^−1^ [1.73 m]^−2^ or UACR ≤33.9 mg/mmol (≤300 mg/g) was low, there was no indication that the efficacy of dapagliflozin differed in these subgroups compared with the overall population (*p* for interaction >0.38; ESM Fig. 4).

### Safety outcomes

The incidence of discontinuation due to adverse events and serious adverse events was similar in patients treated with dapagliflozin and placebo, and did not vary across subgroups of UACR, eGFR and KDIGO risk categories (all *p* for interaction >0.20, Table 2).

**Table 2:** Safety and adverse events of special interest by baseline KDIGO risk categories and subgroups of UACR and eGFR

	*n*/*N*		OR (95% CI)	*p* for interaction
	Dapagliflozin	Placebo		
Discontinuation due to adverse event				
Overall	118/2149	123/2149	0.97 (0.74, 1.26)	
KDIGO risk category				
Moderately high risk	8/330	12/289	0.56 (0.22, 1.38)	0.41
High risk	31/651	32/696	1.03 (0.62, 1.71)	
Very high risk	79/1168	79/1164	1.03 (0.74, 1.42)	
UACR subgroup				
UACR ≤113.0	55/1102	50/1119	1.12 (0.76, 1.66)	0.48
UACR >113.0 to ≤395.5	47/881	54/882	0.87 (0.58, 1.30)	
UACR >395.5	16/166	19/148	0.71 (0.35, 1.46)	
eGFR subgroup				
eGFR ≥45	30/879	42/901	0.73 (0.45, 1.19)	0.20
eGFR ≥30 to <45	60/977	45/917	1.26 (0.84, 1.87)	
eGFR <30	28/293	36/331	0.84 (0.50, 1.43)	
Any serious adverse event				
Overall	633/2149	729/2149	0.81 (0.72, 0.93)	
KDIGO risk category				
Moderately high risk	89/330	106/289	0.64 (0.45, 0.90)	0.26
High risk	176/651	205/696	0.89 (0.70, 1.12)	
Very high risk	368/1168	418/1164	0.83 (0.70, 0.98)	
UACR subgroup				
UACR ≤113.0	284/1102	344/1119	0.78 (0.65, 0.95)	0.79
UACR >113.0 to ≤395.5	279/881	313/882	0.85 (0.69, 1.03)	
UACR >395.5	70/166	72/148	0.77 (0.49, 1.22)	
eGFR subgroup				
eGFR ≥45	237/879	289/901	0.78 (0.63, 0.96)	0.54
eGFR ≥30 to <45	295/977	302/917	0.88 (0.72, 1.07)	
eGFR <30	101/293	138/331	0.72 (0.52, 1.00)	

Data are based on the safety population of 4298

Cut-off values for UACR are mg/mmol (in mg/g: ≤1000 mg/g, >1000 to ≤3500 mg/g, >3500 mg/g); cut-off values for eGFR are ml min^−1^ [1.73 m]^−2^

## Discussion

The benefits of dapagliflozin on kidney and cardiovascular endpoints in patients with CKD are present across all assessed KDIGO risk categories, as defined by UACR and/or eGFR, without evidence of heterogeneity. The absolute benefits of dapagliflozin were consistent across KDIGO risk categories, but markedly increased among patients with higher levels of UACR. These findings are evident among participants with and without type 2 diabetes. Together, these findings support the initiation of dapagliflozin treatment in a wide group of patients with CKD irrespective of baseline KDIGO risk or presence of diabetes.

The DAPA-CKD trial was preceded by a cardiovascular endpoint trial, Dapagliflozin Effect on Cardiovascular Events –Thrombolysis in Myocardial Infarction 58 (DECLARE-TIMI 58), which randomised 17,160 patients with type 2 diabetes at high cardiovascular risk and early-stage CKD. In this trial, dapagliflozin significantly reduced the risk of the co-primary endpoint of heart failure hospitalisation or cardiovascular death and the secondary kidney endpoint [10]. Post hoc analyses from DECLARE-TIMI 58 demonstrated that patients with lower eGFR and higher albuminuria experienced a higher rate of progressive kidney disease, but the benefits of dapagliflozin in reducing the risk of kidney and heart failure outcomes were evident irrespective of baseline UACR and eGFR [11–13]. In the current study, we extend these observations from DECLARE-TIMI 58, and show that the risk of kidney failure and cardiovascular outcomes continues to increase in patients with albuminuria levels well above 300 mg/g and in patients without type 2 diabetes. Taken together, the data from the DECLARE-TIMI 58 and DAPA-CKD trials provide compelling evidence to initiate treatment with SGLT2 inhibitors to prevent kidney failure, heart failure hospitalisations or cardiovascular death in patients with type 2 diabetes regardless of kidney function or albuminuria status.

Other outcome trials comparing SGLT2 inhibitors to placebo also reported no effect modification by baseline KDIGO risk classification on kidney and cardiovascular benefits of SGLT2 inhibitors. These trials, Empagliflozin, Cardiovascular Outcomes, and Mortality in Type 2 Diabetes (EMPA-REG OUTCOME), Canagliflozin Cardiovascular Assessment Study and CANVAS-Renal (CANVAS Program) and Evaluation of Ertugliflozin Efficacy and Safety Cardiovascular Outcomes Trial (VERTIS CV), enrolled patients with type 2 diabetes who were at high risk for cardiovascular disease, most of whom had normal, or near normal, kidney function [14–16]. In line with our results, these trials demonstrated that the relative effects of SGLT2 inhibitors were consistent regardless of KDIGO risk category [17–19]. In conjunction with the results from the current study, these data indicate that the benefit of SGLT2 inhibitors is not affected by kidney disease severity.

The DAPA-CKD trial enrolled patients with CKD with and without type 2 diabetes [4]. Previous studies reported that dapagliflozin consistently reduced the risk of kidney and cardiovascular endpoints in patients with and without type 2 diabetes irrespective of the underlying cause of kidney disease [6]. In the current study, we demonstrated that, in patients without type 2 diabetes, the effect of dapagliflozin on kidney and cardiovascular outcomes is consistent across KDIGO risk categories and the spectrum of UACR and eGFR values included. Specifically, the consistent effects of dapagliflozin across the spectrum of baseline UACR values indicate that, even in patients without diabetes and microalbuminuria (UACR 3.4–33.9 mg/mmol [30–300 mg/g]), dapagliflozin slows progressive kidney function decline. These data thus further support the use of dapagliflozin in a broad range of patients with and without type 2 diabetes to prevent clinically important outcomes.

We also analysed the effect of dapagliflozin on eGFR trajectories according to KDIGO CKD risk categories. By definition, patients with a very high risk score had the lowest eGFR values at baseline. During placebo treatment, eGFR decline during the trial was less pronounced in the highest risk category (−3.5 ml min^−1^ [1.73 m]^−2^ per year) compared with the high risk patients (−4.0 ml min^−1^ [1.73 m]^−2^ per year) or moderately high risk patients (−4.6 ml min^−1^ [1.73 m]^−2^ per year). However, the benefit of dapagliflozin in slowing the progression of eGFR decline was similar across KDIGO risk categories. Consistent with the mechanism of action and prior clinical trials, dapagliflozin reduced eGFR during the first 2 weeks of treatment in all KDIGO risk categories [20]. During chronic treatment, dapagliflozin stabilised the mean eGFR decline in each KDIGO risk category.

More than 300 participants in the DAPA-CKD trial had a UACR above 395.5 mg/mmol (3500 mg/g) at baseline. This subgroup represents highly vulnerable patients in whom blood pressure and HbA_1c_ was significantly higher, while their eGFR was markedly lower than those with less severe albuminuria. The prognosis of these participants was generally poor. Specifically, the event rates for the kidney-specific endpoints and heart failure hospitalisation or cardiovascular death endpoint were approximately ten- and threefold higher, respectively, compared with patients with UACR below 113.0 mg/mmol (1000 mg/g). These data are consistent with the well-known association between UACR and kidney outcomes [8, 21–24]. Similar results were also observed in the CREDENCE trial, with substantially increased rates of kidney and cardiovascular events among patients with type 2 diabetes and CKD and a UACR above 339.0 mg/mmol (3000 mg/g) [25]. Despite the advanced stage of kidney disease and vulnerable population included in the DAPA-CKD trial, adverse and serious adverse event rates were similar with dapagliflozin compared with placebo across all categories of baseline UACR. These data provide reassurance that dapagliflozin treatment may be safely initiated in high-risk patients with severely increased albuminuria.

The results of this study should be interpreted in the context of its limitations. First, the trial was discontinued prematurely at the recommendation of the independent data monitoring committee due to clear efficacy on the primary outcome and survival [4]. This led to a shorter follow-up time and lower number of events than originally planned, limiting the statistical power in certain subgroups, such as patients with albuminuria <33.9 mg/mmol (<300 mg/g) or eGFR > 60 ml min^−1^ [1.73 m]^−2^. However, the consistent benefit for kidney and cardiovascular outcome observed with dapagliflozin in previous cardiovascular outcome trials and in the DAPA-CKD trial suggest that benefits will accrue during prolonged treatment as would occur in clinical practice. The subgroup of patients without diabetes enrolled in the DAPA-CKD trial had CKD with micro- or macroalbuminuria. Whether dapagliflozin reduces the risk of kidney and cardiovascular endpoints in people without diabetes and without albuminuria requires further study. The Study of Heart and Kidney Protection with Empagliflozin (EMPA-KIDNEY) trial is assessing the efficacy of empagliflozin in CKD patients with and without diabetes, and permits inclusion of patients with any level of UACR with an eGFR between 20–45 ml min^−1^ [1.73 m]^−2^ [26].

In conclusion, these findings demonstrate that dapagliflozin reduces the relative risk of kidney and cardiovascular outcomes to a similar extent across subgroups of KDIGO risk categories. These results were consistent in patients with and without type 2 diabetes. Together, these findings support the initiation of dapagliflozin treatment across a wide range of patients with CKD, with and without diabetes, who are at high risk of progressive kidney and cardiovascular disease.

## Supplementary information

ESM 1 (PDF 945 kb)

## Data Availability

Data underlying the findings described in this manuscript may be obtained in accordance with AstraZeneca’s data sharing policy described at: https://astrazenecagrouptrials.pharmacm.com/ST/Submission/Disclosure

## Abbreviations

CKD: Chronic kidney disease
CREDENCE: Canagliflozin and Renal Events in Diabetes With Established Nephropathy Clinical Evaluation
DAPA-CKD: Dapagliflozin and Prevention of Adverse Outcomes in Chronic Kidney Disease
DECLARE-TIMI 58: Dapagliflozin Effect on Cardiovascular Events –Thrombolysis in Myocardial Infarction 58
ESKD: End-stage kidney disease
KDIGO: Kidney Disease Improving Global Outcomes
SGLT2: Sodium–glucose cotransporter 2
UACR: Urinary albumin/creatinine ratio
