# Supplementary material for: Effect of dapagliflozin on kidney and cardiovascular outcomes by baseline KDIGO risk categories: a post hoc analysis of the DAPA-CKD trial
Source: Diabetologia. 2022 Apr 21;65(7):1085–97. doi: 10.1007/s00125-022-05694-6 (PMC9174107; doi:10.1007/s00125-022-05694-6)
Supplement: Supplementary file 1 — (PDF 945 kb) [file 125_2022_5694_MOESM1_ESM.pdf]

ESM Table 1: Baseline characteristics by baseline subgroup of UACR and eGFR

	UACR categories (mg/g)				eGFR categories (ml min^{-1} [1.73 $m]^{-2}$)			
	≤1000	>1000 to ≤3500	>3500	p value*	≥45	≥30 to <45	<30	p value*
Number of patients, n (%)	2225 (51.7%)	1764 (41.0%)	315 (7.3%)		1782 (41.4)	1898 (44.1)	624 (14.5)	
Mean age, years (SD)	62.7 (12.2)	61.0 (12.2)	60.1 (10.7)	<0.001	61.4 (11.8)	62.1 (12.4)	62.3 (12.1)	0.12
Female sex, n (%)	723 (32.5)	581 (32.9)	121 (38.4)	0.11	563 (31.6)	637 (33.6)	225 (36.1)	0.11
Race, n (%)				<0.001				0.08
White	1238 (55.6)	915 (51.9)	137 (43.5)		942 (52.9)	1013 (53.4)	335 (53.7)	
Black	104 (4.7)	75 (4.2)	12 (3.8)		80 (4.5)	88 (4.6)	23 (3.7)	
Asian	760 (34.2)	609 (34.5)	98 (31.1)		590 (33.1)	668 (35.2)	209 (33.5)	
Other	123 (5.5)	165 (9.3)	68 (21.6)		170 (9.5)	129 (6.8)	57 (9.1)	
Mean BMI, kg/m^2 (SD)	29.5 (6.1)	29.6 (6.2)	29.5 (6.3)	0.88	29.5 (6.0)	29.6 (6.2)	29.3 (6.4)	0.59
Current smoker, n (%)	269 (12.1)	264 (15.0)	51 (16.2)	0.03	265 (14.9)	232 (12.2)	87 (13.9)	0.08
Mean blood pressure, mmHg (SD)								
Systolic	134.2 (16.6)	139.3 (17.8)	144.9 (16.7)	<0.001	136.9 (16.8)	136.9 (17.4)	138.2 (18.8)	0.23
Diastolic	76.4 (10.4)	78.6 (10.5)	79.6 (9.7)	<0.001	78.1 (10.1)	77.1 (10.7)	77.1 (11.0)	0.007
Mean eGFR, ml min^{-1} [1.73 $m]^{-2}$ (SD)	44.0 (12.2)	42.5 (12.4)	40.6 (12.4)	<0.001	55.3 (8.1)	37.0 (4.3)	26.8 (1.8)	<0.001
Mean HbA1c, mmol/mol (SD)	53 (18.6)	54 (18.6)	60 (20.8)		56 (19.7)	53 (18.6)	50 (16.4)	
Mean HbA1c, % (SD)	7.0 (1.7)	7.1 (1.7)	7.6 (1.9)	<0.001	7.3 (1.8)	7.0 (1.7)	6.7 (1.5)	<0.001
Haemoglobin, g/l (SD)	130 (17)	128 (19)	121 (18)	<0.001	133 (18)	126 (17)	120 (16)	<0.001

Median UACR, mg/g (IQR)	488 (326–697)	1744 (1310–2394)	4127 (3810–4628)	<0.001	856 (434–1710)	962 (491–1926)	1235 (588–2376)	<0.001
Median UACR, mg/mmol (IQR)	55.1 (36.8–78.7)	197.1 (148.0–270.5)	466.4 (430.5–523.0)	<0.001	96.7 (49.0–193.2)	108.7 (55.5–217.6)	139.6 (66.4–268.5)	<0.001
Type 2 diabetes, n (%)	1433 (64.4)	1204 (68.2)	269 (85.4)	<0.001	1266 (71.0)	1239 (65.3)	401 (64.3)	<0.001
Cardiovascular disease, n (%)	815 (36.6)	669 (37.9)	126 (40.0)	0.43	669 (37.5)	710 (37.4)	231 (37.0)	0.97
Medications, n (%)								
ACE inhibitor / ARB	2160 (97.1)	1712 (97.0)	302 (95.9)	0.49	1749 (98.1)	1837 (96.8)	588 (94.2)	<0.001
Diuretics	942 (42.3)	782 (44.3)	158 (50.2)	0.03	672 (37.7)	880 (46.4)	330 (52.9)	<0.001
GLP-1 receptor agonists†	53 (3.7)	55 (4.6)	14 (5.2)	0.37	47 (3.7)	60 (4.8)	15 (3.7)	0.33
Mineralocorticoid receptor antagonists	105 (4.7)	101 (5.7)	23 (7.3)	0.10	92 (5.2)	113 (5.9)	24 (3.8)	0.12
Statin, n (%)	769 (34.5)	660 (37.4)	169 (53.6)	<0.001	652 (36.6)	725 (38.2)	221 (35.4)	0.38

ACE = angiotensin-converting enzyme; ARB = angiotensin receptor blocker; BMI = body mass index; eGFR = estimated glomerular filtration rate; GLP-1 = glucagon-like peptide 1; HbA1c = glycated haemoglobin; UACR = urinary albumin:creatinine ratio.

*Statistically significant differences across the 3 strata of baseline UACR or eGFR.

†Only in patients with diabetes (N=2,906)

ESM Fig 1: Effects of dapagliflozin across subgroups of UACR, eGFR and KDIGO risk categories in patients with (a) and without diabetes (b)

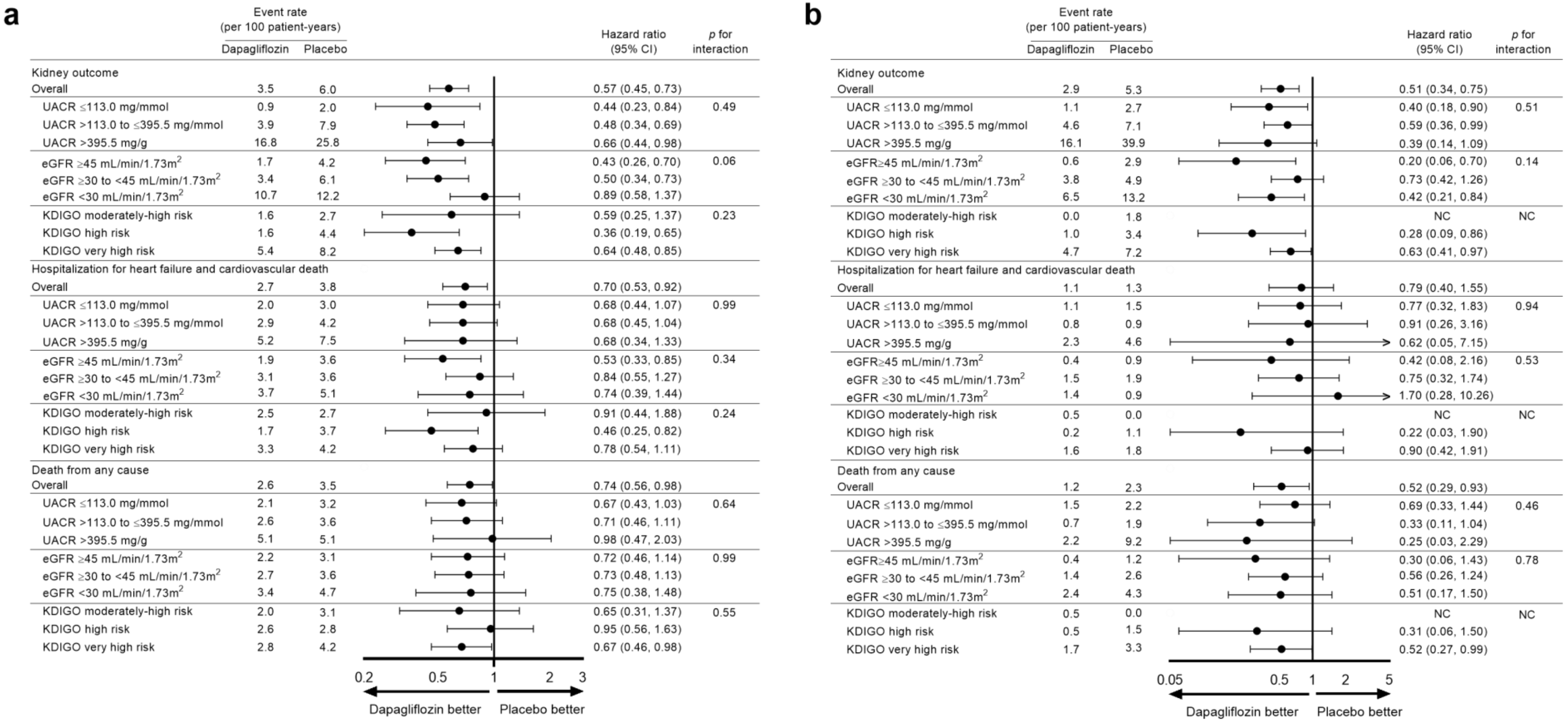

The kidney outcome is a composite of a eGFR decline of at least 50%, end-stage kidney disease or kidney death.

eGFR = estimated glomerular filtration rate; NC = not computed; UACR = urinary albumin-to-creatinine ratio.

ESM Fig 2: Effects of dapagliflozin on eGFR slope (total, acute and chronic) by baseline UACR, KDIGO and eGFR risk categories

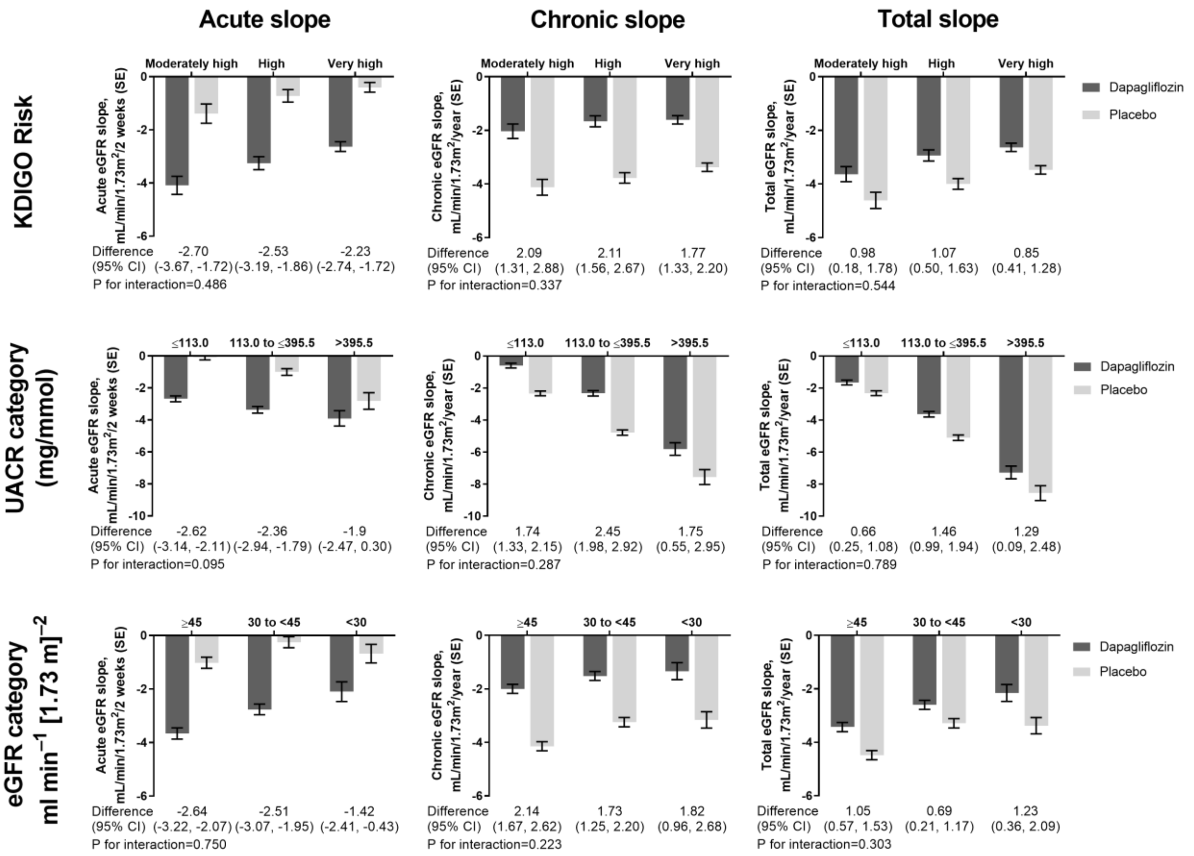

eGFR = estimated glomerular filtration rate; UACR = urinary albumin-to-creatinine ratio

ESM Fig 3: Relative and absolute effects of dapagliflozin on the primary and secondary outcomes across different subgroups of UACR and eGFR. Figure shows the relative effect and absolute risk reduction.

	n/N		Event rate (per 100 patient-years)		Hazard ratio (95% CI)	*p* for interaction	Absolute risk reduction, % (95% CI)	*p* for interaction
	Dapagliflozin	Placebo	Dapagliflozin	Placebo				
**Primary outcome**								
Overall	197/2152	31/2152	4.6	7.5	0.61 (0.51, 0.72)		5.3 (3.4, 7.3)	
UACR ≤113.0 mg/mmol	44/1104	84/1121	1.9	3.7	0.54 (0.37, 0.77)	0.93	3.5 (1.6, 5.4)	0.02
UACR >113.0 to ≤395.5 mg/mmol	97/881	158/883	5.6	9.3	0.59 (0.46, 0.76)		6.9 (3.6, 10.1)	
UACR >395.5 mg/g	56/167	70/148	18.7	31.2	0.61 (0.43, 0.87)		13.8 (3.0, 24.5)	
eGFR ≥45 mL/min/1.73m^2	45/880	95/902	2.4	5.1	0.49 (0.34, 0.69)	0.25	5.4 (2.9, 7.9)	0.88
eGFR ≥30 to <45 mL/min/1.73m^2	93/979	130/919	4.9	7.5	0.61 (0.46, 0.79)		4.6 (1.7, 7.6)	
eGFR <30 mL/min/1.73m^2	59/293	87/331	11.1	14.9	0.73 (0.53, 1.02)		6.1 (-0.5, 12.7)	
**Kidney outcome**								
Overall	142/2152	243/2152	3.3	5.8	0.56 (0.45, 0.68)		4.7 (3.0, 6.4)	
UACR ≤113.0 mg/mmol	21/1104	51/1121	0.9	2.3	0.42 (0.25, 0.70)	0.59	2.6 (1.2, 4.1)	0.002
UACR >113.0 to ≤395.5 mg/mmol	71/881	130/883	4.1	7.7	0.52 (0.39, 0.69)		6.7 (3.7, 9.6)	
UACR >395.5 mg/g	50/167	62/148	16.7	27.7	0.62 (0.42, 0.90)		12.0 (1.4, 22.5)	
eGFR ≥45 mL/min/1.73m^2	26/880	71/902	1.4	3.8	0.38 (0.24, 0.59)	0.10	4.9 (2.8, 7.0)	0.81
eGFR ≥30 to <45 mL/min/1.73m^2	67/979	99/919	3.5	5.7	0.56 (0.41, 0.77)		3.9 (1.4, 6.5)	
eGFR <30 mL/min/1.73m^2	49/293	73/331	9.2	12.5	0.71 (0.49, 1.02)		5.3 (-0.9, 11.5)	
**Hospitalization for heart failure and cardiovascular death**								
Overall	100/2152	138/2152	2.2	3.0	0.71 (0.55, 0.92)		1.8 (0.4, 3.1)	
UACR ≤113.0 mg/mmol	41/1104	59/1121	1.7	2.5	0.70 (0.47, 1.05)	0.98	1.5 (-0.2, 3.3)	0.87
UACR >113.0 to ≤395.5 mg/mmol	42/881	59/883	2.2	3.2	0.70 (0.47, 1.04)		1.9 (-0.3, 4.1)	
UACR >395.5 mg/g	17/167	20/148	4.8	7.0	0.67 (0.35, 1.28)		3.3 (-3.8, 10.5)	
eGFR ≥45 mL/min/1.73m^2	29/880	56/902	1.5	2.8	0.52 (0.33, 0.82)	0.28	2.9 (0.9, 4.9)	0.36
eGFR ≥30 to <45 mL/min/1.73m^2	53/979	58/919	2.6	3.1	0.82 (0.56, 1.19)		0.9 (-1.2, 3.0)	
eGFR <30 mL/min/1.73m^2	18/293	24/331	2.9	3.6	0.83 (0.45, 1.53)		1.1 (-2.8, 5.0)	
**Death from any cause**								
Overall	101/2152	146/2152	2.2	3.2	0.69 (0.53, 0.88)		2.1 (0.7, 3.5)	
UACR ≤113.0 mg/mmol	46/1104	70/1121	1.9	2.9	0.68 (0.47, 0.98)	0.83	2.1 (0.2, 3.9)	0.95
UACR >113.0 to ≤395.5 mg/mmol	38/881	59/883	2.0	3.1	0.64 (0.43, 0.96)		2.4 (0.2, 4.5)	
UACR >395.5 mg/g	17/167	17/148	4.7	5.7	0.83 (0.42, 1.63)		1.3 (-5.6, 8.2)	
eGFR ≥45 mL/min/1.73m^2	34/880	52/902	1.7	2.5	0.67 (0.43, 1.03)	0.99	1.9 (-0.1, 3.9)	0.91
eGFR ≥30 to <45 mL/min/1.73m^2	48/979	63/919	2.3	3.3	0.69 (0.47, 1.00)		2.0 (-0.2, 4.1)	
eGFR <30 mL/min/1.73m^2	19/293	31/331	3.0	4.6	0.68 (0.38, 1.21)		2.9 (-1.3, 7.1)	

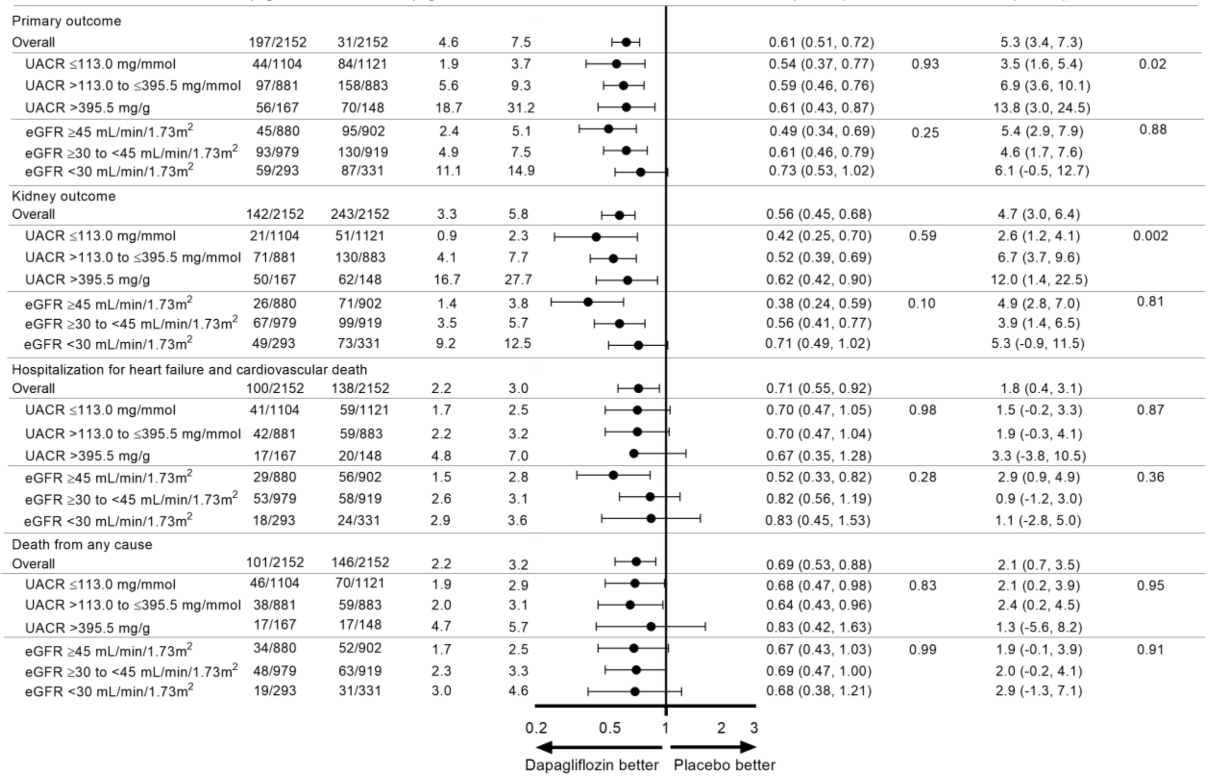

The primary composite outcome is a composite of an eGFR decline of ≥50%, end-stage kidney disease or death from kidney or cardiovascular causes. The kidney composite outcome is a composite of a eGFR decline of ≥50%, end-stage kidney disease or death from kidney causes. eGFR = estimated glomerular filtration rate; UACR = urinary albumin:creatinine ratio.

ESM Fig 4: Effects of dapagliflozin on the primary composite outcome in patients with an UACR above and below 33.9 mg/mmol (300 mg/g) and eGFR above and below 60 mL/min/1.73m^2

	n/N		Event rate (per 100 patient-years)		Hazard ratio (95% CI)	*p* for interaction	Absolute risk reduction, % (95% CI)
	Dapagliflozin	Placebo	Dapagliflozin	Placebo			
Overall	197/2152	312/2152	4.6	7.5	0.61 (0.51, 0.72)		5.3 (3.4, 7.3)
UACR ≤33.9 mg/mmol	9/240	9/205	1.8	2.1	0.86 (0.34, 2.16)	0.38	0.6 (-3.1, 4.3)
UACR >33.9 mg/mmol	188/1912	303/1947	5.0	8.1	0.61 (0.51, 0.73)		5.7 (3.6, 7.8)
eGFR ≥60 mL/min/1.73m^2	15/234	18/220	2.7	3.6	0.75 (0.38, 1.48)	0.58	1.8 (-3.0, 6.6)
eGFR <60 mL/min/1.73m^2	182/1918	294/1932	4.9	8.0	0.60 (0.50, 0.72)		5.7 (3.7, 7.8)

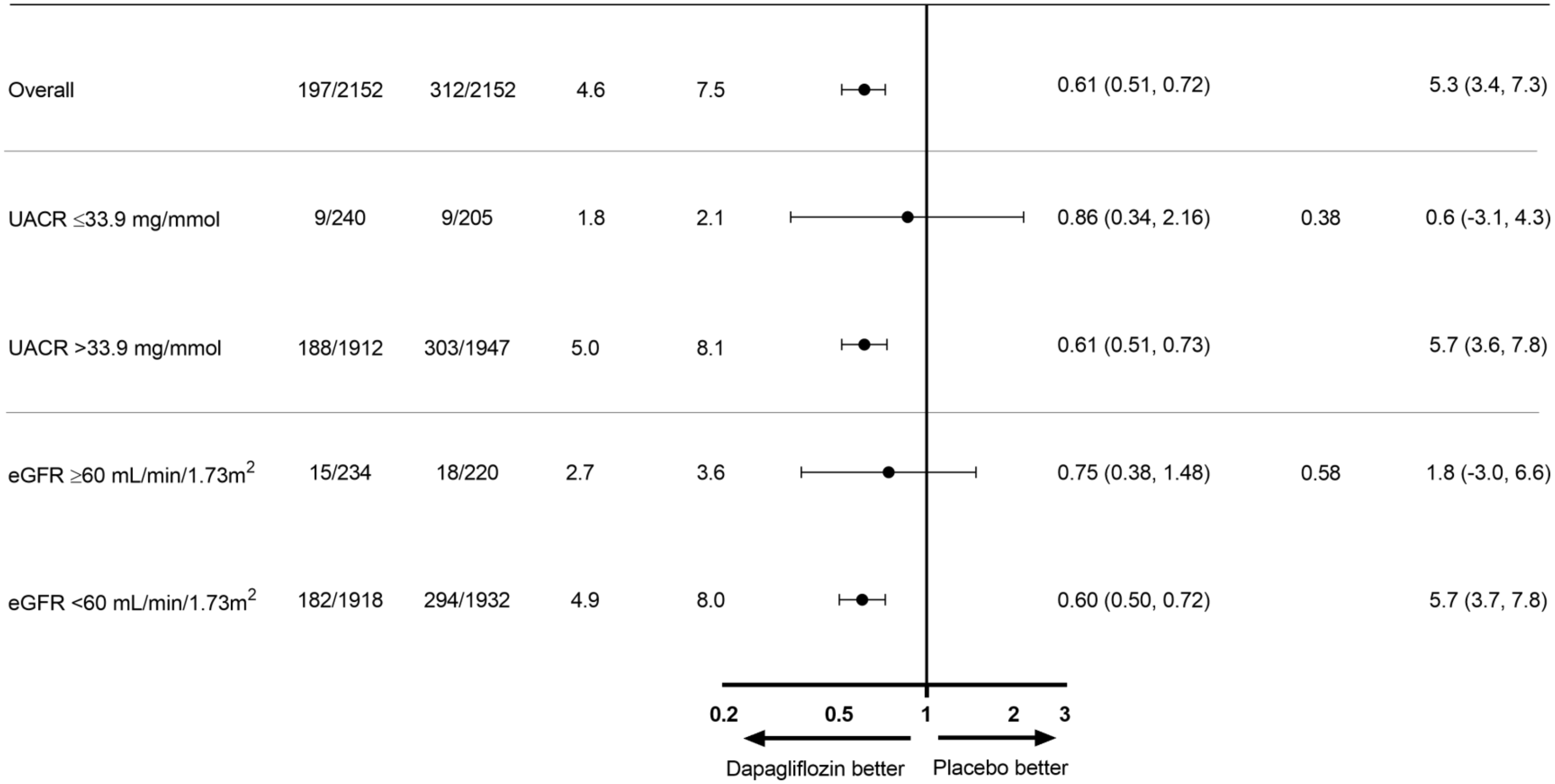

eGFR = estimated glomerular filtration rate; UACR = urinary albumin:creatinine ratio.
